# Supplementary material for: Unveiling the Bioactive Potential of the Invasive Jellyfish Phyllorhiza punctata Through Integrative Transcriptomic and Proteomic Analyses
Source: Biomolecules. 2025 Aug 4;15(8):1121. doi: 10.3390/biom15081121 (PMC12383608; doi:10.3390/biom15081121)
Supplement: Supplementary file 1 [file biomolecules-15-01121-s001.zip › Suplementary Material/FileS3/PEPFOLD4/XP_065066755_359/clusters.html]

 PEP-FOLD clusters


Download archive of all models  here   

|  |  |  |
| --- | --- | --- |
| # | file | sOPEP |
| # cluster 1 - Size: 5 |  |  |
|  | XP\_065066755\_359-model1 | -6.43277 |
|  | XP\_065066755\_359-model1.1 | -6.23545 |
|  | XP\_065066755\_359-model1.2 | -6.00745 |
|  | XP\_065066755\_359-model1.3 | -5.1225 |
|  | XP\_065066755\_359-model1.4 | -4.89341 |
| # cluster 2 - Size: 1 |  |  |
|  | XP\_065066755\_359-model2 | -5.72054 |
| # cluster 3 - Size: 3 |  |  |
|  | XP\_065066755\_359-model3 | -5.1999 |
|  | XP\_065066755\_359-model3.1 | -4.87151 |
|  | XP\_065066755\_359-model3.2 | -4.85079 |
| # cluster 4 - Size: 21 |  |  |
|  | XP\_065066755\_359-model4 | -5.16322 |
|  | XP\_065066755\_359-model4.1 | -5.12538 |
|  | XP\_065066755\_359-model4.2 | -5.10682 |
|  | XP\_065066755\_359-model4.3 | -5.09324 |
|  | XP\_065066755\_359-model4.4 | -5.06022 |
|  | XP\_065066755\_359-model4.5 | -5.05911 |
|  | XP\_065066755\_359-model4.6 | -5.04373 |
|  | XP\_065066755\_359-model4.7 | -5.00978 |
|  | XP\_065066755\_359-model4.8 | -5.00773 |
|  | XP\_065066755\_359-model4.9 | -5.00106 |
|  | XP\_065066755\_359-model4.10 | -4.9968 |
|  | XP\_065066755\_359-model4.11 | -4.98957 |
|  | XP\_065066755\_359-model4.12 | -4.96354 |
|  | XP\_065066755\_359-model4.13 | -4.94973 |
|  | XP\_065066755\_359-model4.14 | -4.92676 |
|  | XP\_065066755\_359-model4.15 | -4.92336 |
|  | XP\_065066755\_359-model4.16 | -4.91661 |
|  | XP\_065066755\_359-model4.17 | -4.87494 |
|  | XP\_065066755\_359-model4.18 | -4.84562 |
|  | XP\_065066755\_359-model4.19 | -4.80547 |
|  | XP\_065066755\_359-model4.20 | -4.78114 |
| # cluster 5 - Size: 2 |  |  |
|  | XP\_065066755\_359-model5 | -5.15235 |
|  | XP\_065066755\_359-model5.1 | -5.02237 |
| # cluster 6 - Size: 4 |  |  |
|  | XP\_065066755\_359-model6 | -5.12575 |
|  | XP\_065066755\_359-model6.1 | -5.00716 |
|  | XP\_065066755\_359-model6.2 | -4.8366 |
|  | XP\_065066755\_359-model6.3 | -4.81774 |
| # cluster 7 - Size: 9 |  |  |
|  | XP\_065066755\_359-model7 | -5.12091 |
|  | XP\_065066755\_359-model7.1 | -5.05912 |
|  | XP\_065066755\_359-model7.2 | -4.95703 |
|  | XP\_065066755\_359-model7.3 | -4.93617 |
|  | XP\_065066755\_359-model7.4 | -4.92536 |
|  | XP\_065066755\_359-model7.5 | -4.89523 |
|  | XP\_065066755\_359-model7.6 | -4.86837 |
|  | XP\_065066755\_359-model7.7 | -4.85148 |
|  | XP\_065066755\_359-model7.8 | -4.83909 |
| # cluster 8 - Size: 2 |  |  |
|  | XP\_065066755\_359-model8 | -5.1128 |
|  | XP\_065066755\_359-model8.1 | -4.92635 |
| # cluster 9 - Size: 5 |  |  |
|  | XP\_065066755\_359-model9 | -5.10681 |
|  | XP\_065066755\_359-model9.1 | -4.98988 |
|  | XP\_065066755\_359-model9.2 | -4.94845 |
|  | XP\_065066755\_359-model9.3 | -4.90225 |
|  | XP\_065066755\_359-model9.4 | -4.81098 |
| # cluster 10 - Size: 2 |  |  |
|  | XP\_065066755\_359-model10 | -5.08521 |
|  | XP\_065066755\_359-model10.1 | -4.96948 |
| # cluster 11 - Size: 3 |  |  |
|  | XP\_065066755\_359-model11 | -5.06375 |
|  | XP\_065066755\_359-model11.1 | -4.89656 |
|  | XP\_065066755\_359-model11.2 | -4.83332 |
| # cluster 12 - Size: 5 |  |  |
|  | XP\_065066755\_359-model12 | -5.06285 |
|  | XP\_065066755\_359-model12.1 | -5.0316 |
|  | XP\_065066755\_359-model12.2 | -4.84983 |
|  | XP\_065066755\_359-model12.3 | -4.802 |
|  | XP\_065066755\_359-model12.4 | -4.7588 |
| # cluster 13 - Size: 7 |  |  |
|  | XP\_065066755\_359-model13 | -5.04968 |
|  | XP\_065066755\_359-model13.1 | -5.00649 |
|  | XP\_065066755\_359-model13.2 | -5.00329 |
|  | XP\_065066755\_359-model13.3 | -4.96773 |
|  | XP\_065066755\_359-model13.4 | -4.9676 |
|  | XP\_065066755\_359-model13.5 | -4.90467 |
|  | XP\_065066755\_359-model13.6 | -4.90228 |
| # cluster 14 - Size: 6 |  |  |
|  | XP\_065066755\_359-model14 | -5.03696 |
|  | XP\_065066755\_359-model14.1 | -5.02429 |
|  | XP\_065066755\_359-model14.2 | -4.98317 |
|  | XP\_065066755\_359-model14.3 | -4.94342 |
|  | XP\_065066755\_359-model14.4 | -4.91435 |
|  | XP\_065066755\_359-model14.5 | -4.90823 |
| # cluster 15 - Size: 5 |  |  |
|  | XP\_065066755\_359-model15 | -5.02733 |
|  | XP\_065066755\_359-model15.1 | -5.02069 |
|  | XP\_065066755\_359-model15.2 | -4.95207 |
|  | XP\_065066755\_359-model15.3 | -4.91093 |
|  | XP\_065066755\_359-model15.4 | -4.86795 |
| # cluster 16 - Size: 2 |  |  |
|  | XP\_065066755\_359-model16 | -5.00685 |
|  | XP\_065066755\_359-model16.1 | -4.93978 |
| # cluster 17 - Size: 3 |  |  |
|  | XP\_065066755\_359-model17 | -4.99286 |
|  | XP\_065066755\_359-model17.1 | -4.89956 |
|  | XP\_065066755\_359-model17.2 | -4.75427 |
| # cluster 18 - Size: 1 |  |  |
|  | XP\_065066755\_359-model18 | -4.9779 |
| # cluster 19 - Size: 1 |  |  |
|  | XP\_065066755\_359-model19 | -4.95089 |
| # cluster 20 - Size: 3 |  |  |
|  | XP\_065066755\_359-model20 | -4.94226 |
|  | XP\_065066755\_359-model20.1 | -4.93281 |
|  | XP\_065066755\_359-model20.2 | -4.90668 |
| # cluster 21 - Size: 4 |  |  |
|  | XP\_065066755\_359-model21 | -4.93911 |
|  | XP\_065066755\_359-model21.1 | -4.89817 |
|  | XP\_065066755\_359-model21.2 | -4.89761 |
|  | XP\_065066755\_359-model21.3 | -4.84656 |
| # cluster 22 - Size: 4 |  |  |
|  | XP\_065066755\_359-model22 | -4.93822 |
|  | XP\_065066755\_359-model22.1 | -4.86938 |
|  | XP\_065066755\_359-model22.2 | -4.86693 |
|  | XP\_065066755\_359-model22.3 | -4.82426 |
| # cluster 23 - Size: 2 |  |  |
|  | XP\_065066755\_359-model23 | -4.88354 |
|  | XP\_065066755\_359-model23.1 | -4.86005 |
|  |  |  |
